# Magnitude of undernutrition and associated factors among patients with tuberculosis in the Benadir Region, Somalia: A multicenter cross-sectional study

**DOI:** 10.1016/j.ijregi.2026.100949

**Published:** 2026-07-15

**Authors:** Jamal Hassan Mohamoud, Mohamed Hussein Adam, Ahmed Mahad Sheikh Mohamed, Bashiru Garba, Mohamed Adam Mahamud, Hodo Aideed Asowe, Samira Abdullahi Moalim, Najma Abdiwahab Shirwa, Aniso Hassan Mohamud

**Affiliations:** 1 Department of Public Health, Faculty of Medicine and Health Sciences, SIMAD University, Mogadishu, Somalia; 2 SIMAD Institute for Global Health, SIMAD University, Mogadishu, Somalia; 3 Department of Microbiology and Laboratory Science, Faculty of Medicine and Health Sciences, SIMAD University, Mogadishu, Somalia; 4 Department of Nursing and Midwifery, Faculty of Medicine and Health Sciences, SIMAD University, Mogadishu, Somalia; 5 Faculty of Bioscience Engineering, Ghent University, The International Training Centre, Ghent, Belgium; 6 Department of Applied Human Nutrition, Faculty of Agriculture, University of Nairobi, Nairobi, Kenya

**Keywords:** Tuberculosis, Undernutrition, Nutritional status, Treatment adherence, Body mass index

## Abstract

**Objectives:**

Tuberculosis remains a major public health problem in low- and middle-income countries and is closely associated with poor nutritional status among patients. Undernutrition in patients with tuberculosis contributes to adverse treatment outcomes, increased morbidity, and delayed recovery. However, evidence on the magnitude of undernutrition and its associated factors among patients with tuberculosis in Somalia is limited. This study aimed to assess the prevalence of undernutrition and identify associated factors among adults with tuberculosis.

**Methods:**

An institution-based cross-sectional study was conducted among 362 patients with tuberculosis who attended selected tuberculosis treatment facilities. Data were collected using a structured questionnaire covering sociodemographic, household, dietary, clinical, and treatment-related factors. Anthropometric measurements were obtained using standardized procedures, and body mass index was calculated to assess nutritional status. Undernutrition was defined as a body mass index of <18.5 kg/m². Bivariate and multivariable logistic regression analyses were performed to identify factors associated with undernutrition, and adjusted odds ratios (AORs) and 95% confidence intervals (CIs) were reported.

**Results:**

The prevalence of undernutrition among patients with tuberculosis was 37.6% (95% CI, 31.5–41.2). In the multivariable analysis, female sex (AOR = 3.93; 95% CI, 1.84–4.81), household size of 5 or more members (AOR = 3.15; 95% CI, 1.92–5.17), rural residence (AOR = 5.49; 95% CI, 3.16–9.28), extrapulmonary tuberculosis (AOR = 3.56; 95% CI, 1.93–6.27), continuation phase of treatment (AOR = 3.10; 95% CI, 1.92–4.11), treatment duration (AOR = 3.82; 95% CI, 2.09–5.30), and poor treatment adherence (AOR = 4.71; 95% CI, 2.81–8.02) were significantly associated with undernutrition.

**Conclusion:**

Undernutrition was highly prevalent among adults with tuberculosis. Both sociodemographic and treatment-related factors were independently associated with undernutrition. These findings highlight the importance of integrating routine nutritional assessments and targeted nutritional support into tuberculosis care services.

## Highlights

• First study assessing undernutrition among patients with Tuberculosis (TB) in Benadir, Somalia.
• Undernutrition affected 37.6% of adults receiving TB treatment.
• Female sex, rural residence, and extrapulmonary TB increased undernutrition risk.
• Poor treatment adherence was strongly associated with undernutrition.
• Routine nutrition screening should be integrated into TB care services.

## Introduction

Tuberculosis (TB) is a contagious illness that impacts millions globally. In the last 2 years, approximately 10 million individuals worldwide have contracted TB, and around 1.4 million people have died from it. Notably, 95% of these cases are found in developing countries [1,2]. Despite this, TB is both curable and preventable. Around 85% of those who develop the disease can be effectively treated with a 6-month course of medication, and treatment durations ranging from 1 to 6 months are available for TB infection [3]. Evidence suggests a reciprocal link between TB and undernutrition, with a significant number of those with active TB also facing weight loss [4]. This nutritional deficiency is associated with an increased occurrence and intensity of illnesses in these patients, leading to a two-fold to three-fold rise in their mortality rates [5]. Individuals diagnosed with TB typically have a lower body mass index (BMI), diminished muscle mass, and less subcutaneous fat compared with the general population and they are 11 times more likely to possess a BMI below 18.5 [6].

Somalia continues to bear a high burden of TB, despite program progress in recent years. According to the World Health Organization, TB incidence in Somalia was estimated at approximately 246 cases per 100,000 population in 2023, having declined modestly from about 286 per 100,000 in 2010 [7]. However, this burden is sustained within a context of protracted humanitarian crisis characterized by decades of conflict, recurrent climate shocks, mass displacement, and chronic food insecurity. These overlapping crises have eroded household livelihoods, disrupted food systems, and severely constrained access to diverse and adequate diets, resulting in widespread undernutrition across all age groups.

Socioeconomic, behavioral, and clinical factors are commonly associated with undernutrition in individuals undergoing TB treatment. In particular, patients from low-income households are at a higher risk of undernutrition [8]. Unemployment, larger family sizes, and household food insecurity are strongly associated with an increased risk of undernutrition.[9] In addition, literacy status and gender have been linked to undernutrition during TB infection, with lower educational attainment and female sex being associated with a higher likelihood of undernutrition [10].

To our knowledge, there is no published evidence on undernutrition among patients with TB in Somalia, particularly at the regional and facility levels. Understanding the extent of undernutrition and identifying its key determinants among patients with TB is essential for informing targeted nutritional interventions and strengthening integrated TB care services. Therefore, this study aimed to assess the magnitude of undernutrition and its associated factors among patients with TB in the Benadir region of Somalia.

## Methods

### *Study design, setting, and duration*

An institution-based cross-sectional study was conducted to determine the magnitude of undernutrition and its associated factors among patients with TB. The study was carried out in selected TB treatment facilities in the Benadir region of Somalia, namely, Manhal, Sacid, Ayan, and Dharkenley health facilities, which provide routine TB diagnosis, treatment, and follow-up services as part of the national TB control efforts. These facilities serve large catchment populations in urban Mogadishu and the surrounding districts.

### *Study population and eligibility criteria*

The study included adult patients with TB (≥18 years) attending Manhal, Sacid, Ayan, and Dharkenley health facilities in the Benadir region of Somalia during the study period (September 2 to December 12, 2025). Patients receiving anti-TB treatment who consented to participate were included. Patients who were severely ill or had conditions that affected accurate anthropometric measurements were excluded.

### *Sample size determination*

The sample size was determined using the single population proportion formula (n = [z α/2]^2^ * p[1-p]/ d²). A study from Ethiopia [11] reported that 38.1% of patients with TB were malnourished, which was used as the prevalence rate. The margin of error was set at 0.05 with a confidence level of 95%. This resulted in a required sample size of 362 participants.

### *Sample technique procedure*

A facility-based systematic sampling technique was employed. The total sample size was proportionally allocated to the selected health facilities based on the average TB patient load during the study period. A sampling interval of k = 4 was used, and every fourth eligible patient with TB attending routine treatment initiation or follow-up visits was recruited until the required sample size was achieved.

### *Data collection procedures*

Data collection was conducted using a structured questionnaire, which was crafted based on a thorough review of relevant literature [12,13]. This questionnaire gathered information on various aspects, including sociodemographic, household, dietary, clinical, and treatment-related characteristics. The Morisky Medication Adherence Scale-8 was used to assess adherence to TB treatment, with a score range of 0 to 8. A score greater than 8 signified high adherence, scores between 6 and 8 indicated medium adherence, and scores below 6 reflected low adherence [14,15]. The questionnaire was first created in English, then translated into Somali, and subsequently back-translated into English to ensure accuracy. Data collectors underwent a 3-day training session covering study procedures, ethical issues, and data quality assurance. A pretest involving 5% of participants from outside the study locations was conducted, and necessary adjustments were made before the actual data collection began.

### *Variables and measurements*

The outcome of interest was nutritional status, classified as undernutrition (BMI <18.5 kg/m²) or normal nutritional status (BMI ≥18.5 kg/m²). The independent factors included sociodemographic factors (age, sex, marital status, educational attainment, occupational status, family size, and place of residence), household and dietary factors (household size, access to clean drinking water and sanitation facilities, household smoke exposure, household ventilation status, number of meals consumed per day, number of food groups consumed per day, and food preference status), and clinical and treatment-related factors (type of TB, phase of TB treatment, duration of anti-TB treatment, presence of chronic comorbid conditions, unexpected weight loss, appetite status, and treatment adherence).

#### Anthropometric measurements

Anthropometric measurements, including weight and height, were performed using standardized procedures. Body weight was measured to the nearest 0.1 kg using a calibrated digital scale (German Seca model), with the participants wearing light clothing and no shoes. Height was measured to the nearest 0.1 cm using a height-measuring board, with participants standing upright against a wall without shoes, heels together, head positioned in the Frankfurt plane, and their eyes looking straight ahead. Treatment adherence was assessed using the Morisky Medication Adherence Scale-8, and categorized as poor (<6), medium (6–7), and high adherence (8). For analytical purposes, adherence was dichotomized into poor and good adherence. Treatment duration was categorized as <3 months and ≥3 months based on clinical relevance and treatment phases.

#### BMI

BMI was calculated by dividing body weight in kilograms by the square of height in meters (kg/m²). Severe undernutrition was defined as a BMI <16.0 kg/m², moderate undernutrition as 16.0–16.99 kg/m², and mild undernutrition as 17.0–18.49 kg/m². Normal weight was defined as a BMI of 18.5–24.99 kg/m², overweight as 25.0–29.99 kg/m², and obesity as ≥30.0 kg/m². For analytical purposes, BMI was further categorized into two groups: undernutrition (BMI <18.5 kg/m²) and normal nutritional status (BMI ≥18.5 kg/m²).

### *Data quality assurance*

To maintain high data quality, several strategies were put in place. Data collectors received close supervision during the entire data gathering phase. Each day, completed questionnaires were reviewed to ensure they were complete and consistent. Any information that was missing or unclear was quickly addressed by consulting medical records or seeking clarification from participants when necessary.

### *Statistical analysis*

Data were entered, cleaned, and analyzed using SPSS software (version 29.0). Descriptive statistics were used to outline the participants’ characteristics of the study. Bivariate logistic regression was conducted to identify candidate variables for multivariable analysis. Variables with a *P* value of ≤ 0.20 were considered for inclusion; however, selection was not based solely on statistical significance. Variables were also included in the multivariable model based on prior evidence from the literature and their theoretical relevance as potential confounders. Adjusted odds ratios (AORs) and 95% confidence intervals (CIs) were calculated, with statistical significance set at *P* ≤ 0.05. The Hosmer-Lemeshow goodness-of-fit test was used to assess model adequacy. Additionally, the variance inflation factor was used to detect multicollinearity among independent variables. Given the relatively high prevalence of undernutrition in this study, the use of odds ratios may overestimate the strength of associations compared with risk ratios. Alternative models such as log-binomial and Poisson regression with robust variance were considered; however, logistic regression was retained due to model stability and consistency with prior literature. Therefore, the findings should be interpreted with caution.

### *Ethics approval and consent to participate*

This study was conducted in accordance with the Declaration of Helsinki [16]. Ethical approval was obtained from the Institutional Review Board of the Faculty of Medicine and Health Sciences at SIMAD University (Ref: 2025/SU-IRB/FMHS/P0090). Written informed consent was obtained from all participants before participation, and the confidentiality of the collected data was strictly maintained.

## Results

### *Sociodemographic characteristics*

A total of 362 adults with TB were included in the study. The sociodemographic characteristics of the participants are presented in Table 1. The mean age was 35.2 ± 14.0 years, with the majority of participants aged <40 years (72.4%). Males constituted 74.0% of the study population. More than half of the participants were married (51.1%), and most had no formal education (60.8%). Unemployment was common (71.0%), and most participants resided in urban areas (64.1%).

**Table 1:** Sociodemographic characteristics of adult patients with tuberculosis in the Benadir Region, Somalia.

Variables	Frequency	Percent
**Age of participants, 35.2 ± 14.0**		
<40 years	262	72.4
40–65 years	89	24.6
>60 years	11	3.0
**Sex**		
Male	268	74.0
Female	94	26.0
**Marital status**		
Currently married	185	51.1
Currently unmarried	177	48.9
**Educational status**		
Informal	220	60.8
Primary	66	18.2
Secondary	43	11.9
Higher education	33	9.1
**Current occupational status**		
Employed	105	29.0
Unemployed	257	71.0
**Residence**		
Urban	232	64.1
Rural	130	35.9
**Family size**		
<5	126	34.8
≥5	236	65.2

### *Household characteristics and dietary practices*

Household characteristics and dietary practices are summarized in Table 2. Most participants (63.8%) lived in households with 5 or more household members. Access to clean drinking water and sanitation facilities was reported by 90.6% and 88.7% of the households, respectively. Household smoke exposure was reported by 27.6% of participants. Most participants consumed 1 to 3 meals per day (87.0%), and 66.0% reported consuming 1 to 2 food groups per day.

**Table 2:** Household characteristics and dietary practices among adult patients with tuberculosis in the Benadir Region, Somalia.

Variables	Frequency	Percent
**Household member**		
<5	131	36.2
≥5	231	63.8
**Access to clean drinking water**		
Yes	328	90.6
No	34	9.4
**Access to proper sanitation facilities**		
Yes	321	88.7
No	41	11.3
**Smoke in household**		
Yes	100	27.6
No	262	72.4
**Household ventilation status**		
Yes	304	84.0
No	58	16.0
**Meal per day**		
1–3	315	87.0
>3	47	13.0
**Number of food groups per day**		
1–2	239	66.0
>2	123	34.0
**Food preference status**		
Yes	221	61.0
No	141	39.0

### *Clinical and treatment-related characteristics*

The clinical and treatment-related characteristics are presented in Table 3. Pulmonary TB was the most common disease (62.7%). More than half of the participants were in the intensive phase of treatment (54.4%). Approximately one-quarter (22.9%) of participants reported having a chronic comorbid condition. Unexpected weight loss was reported by 63.8% of the participants, and 39.0% reported a poor appetite. Two-thirds of the participants (66.3%) demonstrated good treatment adherence.

**Table 3:** Clinical and treatment-related characteristics of adult patients with TB in the Benadir Region, Somalia.

Variables	Frequency	Percent
**Category of TB**		
Pulmonary TB	227	62.7
Extrapulmonary TB	135	37.3
**Phase of TB treatment**		
Intensive phase	197	54.4
Continuation phase	165	45.6
**Duration of TB treatment (in month)**		
<3	120	33.1
≥3	242	66.9
**Chronic disease status**		
Yes	83	22.9
No	279	77.1
**Unexpected weight loss**		
Yes	231	63.8
No	131	36.2
**Appetite status**		
Yes	221	61.0
No	141	39.0
**Treatment adherence status**		
Poor	122	33.7
Good	240	66.3

Abbreviation: TB, tuberculosis.

### *Magnitude of undernutrition status*

**Figure 1:**
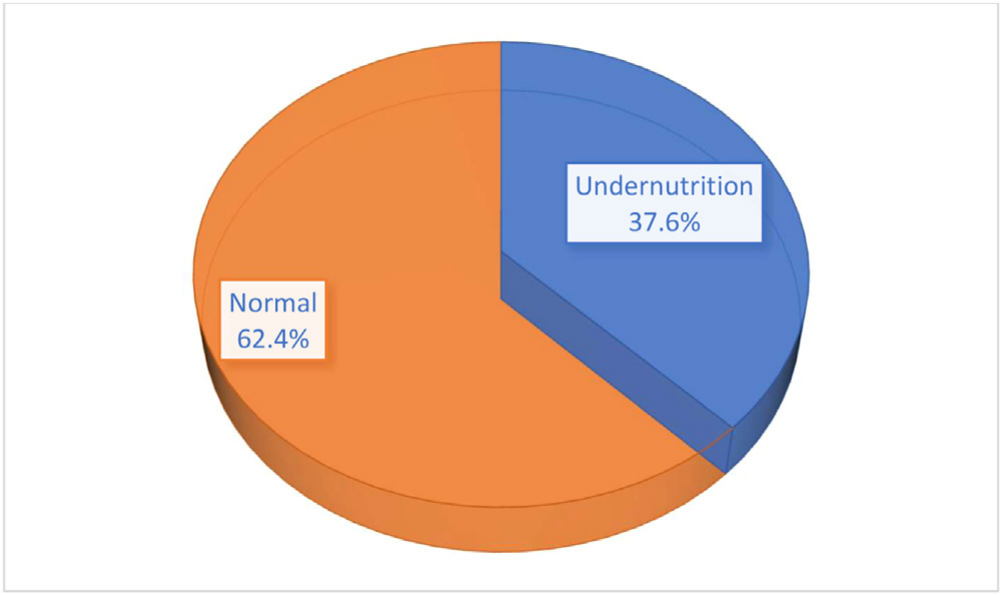
Magnitude of undernutrition among patients with tuberculosis in the Benadir region, Somalia. Undernutrition was defined as body mass index <18.5 kg/m². The figure shows that 37.6% of participants were undernourished, whereas 62.4% had normal nutritional status.

As shown in the Figure 1, more than one-third of the patients with TB were undernourished, with an overall prevalence of 37.6%, while 62.4% had a normal nutritional status.

### *Associated factors with undernutrition*

In the bivariate logistic regression analysis, several sociodemographic, household, and clinical variables were associated with undernutrition at a *P* value of ≤ 0.20 and were included in the multivariable model. These variables included sex, marital status, occupational status, family size, place of residence, type of TB, phase of TB treatment, duration of treatment, and treatment adherence (Table 4). In the final multivariable logistic regression model, female sex was independently associated with undernutrition, with females having higher odds compared with males (AOR = 3.93; 95% CI, 1.84–4.81). Patients from households with 5 or more members were more likely to be undernourished than those from smaller households (AOR = 3.15; 95% CI, 1.92–5.17). Rural residence was significantly associated with undernutrition (AOR = 5.49; 95% CI, 3.16–9.28).

**Table 4:** Bivariate and multivariable logistic regression analysis of factors associated with undernutrition among adult patients with tuberculosis in the Benadir Region, Somalia.

Variables	Undernutrition status		COR (95% CI)	AOR (95% CI)	*P* value
	Yes	No			
**Sex**					
Male	85 (62.5)	183 (81.0)	Ref		
Female	51 (37.5)	43 (19.0)	3.82 (1.57–4.13)	3.93 (1.84–4.81)	<0.001^a^
**Marital status**					
Married	57 (41.9)	128 (56.6)	Ref		
Unmarried	79 (58.1)	98 (43.4)	1.82 (1.17–2.78)	1.31 (0.73–2.59)	0.076
**Occupational status**					
Employed	61 (44.9)	44 (19.5)	2.65 (1.67–4.21)	2.39 (1.47–3.91)	0.021^a^
Unemployed	95 (55.1)	182 (80.5)	Ref		
**Family size**					
<5	33 (24.3)	93 (41.2)	Ref		
≥ 5	103 (75.7)	133 (58.8)	2.18 (1.35–3.50)	3.15 (1.92–4.17)	0.001^a^
**Residence**					
Rural	86 (63.2)	44 (19.5)	7.11 (4.41–11.49)	5.49 (3.16–9.28)	0.024^a^
Urban	50 (36.8)	182 (80.5)	Ref		
**Type of TB**					
Pulmonary TB	101 (74.3)	126 (55.8)	Ref		
Extrapulmonary TB	35 (25.7)	100 (44.2)	2.28 (1.43–3.65)	3.56 (1.93–6.27)	0.001^a^
**Phases of TB treatment**					
Intensive phase	96 (69.9)	101 (44.7)	Ref		
Continuation phase	41 (30.1)	125 (55.3)	2.90 (1.84–4.54)	3.10 (1.92–4.11)	<0.001^a^
**Length of being on treatment**					
<3 Month	71 (52.2)	49 (21.7)	3.94 (2.49–6.26)	3.82 (2.09–5.30)	0.005^a^
≥3 Month	65 (47.8)	177 (78.3)	Ref		
**Adherence treatment status**					
Poor	79 (58.1)	43 (19.0)	5.89 (3.67–9.49)	4.71 (2.81–8.02)	<0.001^a^
Good	57 (41.9)	183 (81.0)	Ref		

Abbreviations: AOR, adjusted odds ratio; CI, confidence interval; COR, crude odds ratio; Ref, reference category; TB, tuberculosis.

a Statistically significant.

Patients with extrapulmonary TB had higher odds of undernutrition than those with pulmonary TB (AOR = 3.56; 95% CI, 1.93–6.27). Undernutrition was more prevalent among patients in the continuation phase of anti-TB treatment than among those in the intensive phase (AOR = 3.10; 95% CI, 1.92–4.11). Additionally, patients who had been on treatment for less than 3 months had higher odds of undernutrition than those treated for 3 months or longer (AOR = 3.82; 95% CI, 2.09–5.30). Poor treatment adherence was strongly associated with undernutrition (AOR = 4.71; 95% CI, 2.81–8.02).

## Discussion

This study assessed the magnitude of undernutrition and its associated factors in adults with TB. The magnitude of undernutrition was 37.6%, indicating that more than one-third of patients receiving TB care were affected by poor nutritional status. This finding highlights the substantial burden of undernutrition among patients with TB and underscores the importance of integrating nutritional assessments into routine TB management. Additionally, the use of odds ratios in a relatively common outcome should be interpreted cautiously, as they may overestimate associations.

The magnitude of undernutrition observed in this study is comparable to findings from facility-based studies conducted in Burkina Faso 35.8% [17], and Bangabandhu, Bangladesh 36% [18]. However, the prevalence reported in this study was lower than that in Haramaya district, Eastern Ethiopia (40.1%) [19], and Adama, central Ethiopia (53%) [20]. Unlike studies conducted in relatively stable settings, Somalia’s protracted humanitarian crisis likely amplifies baseline undernutrition before TB diagnosis. Similarly, these variations may also reflect differences in study populations, with higher-risk groups such as internally displaced persons and urban poor more represented in our study. Moreover, variations in participant sociodemographic and timing and locations of the studies, differences in lifestyle and dietary practices, and the range of sample sizes utilized could all contribute to these discrepancies.

Although males constituted the majority of the study population and had higher levels of formal education, the multivariable logistic regression analysis indicated that female patients had significantly higher odds of undernutrition compared with males (AOR = 3.93; 95% CI, 1.84–4.81). This finding suggests that sex differences in undernutrition among patients with TB persist even after adjusting for other measured sociodemographic factors. The higher risk observed among women may reflect sex-specific vulnerabilities to nutritional depletion during TB illness that are not fully captured by education or employment status alone. Importantly, this result highlights that female patients with TB represent a particularly vulnerable subgroup for undernutrition in this setting, underscoring the need for targeted nutritional assessment and support within TB care services. Additionally, in the African context, more female patients were excluded from socioeconomic and health opportunities, which may result in less access to adequate water, sanitation, balanced diet, and medical care.

In this study, it was observed that adult patients with TB living in households with more than 5 members had a 3.15 times greater likelihood of experiencing undernutrition. This is in agreement with findings from a study in Hosanna [21]. Larger households may face greater challenges in meeting the nutritional needs of all members, particularly in resource-limited settings.

The odds of undernutrition were significantly higher among patients with TB residing in rural areas compared with those living in urban areas. This finding is consistent with a previous study conducted in South Ethiopia region [22]. This may be explained by limited access to healthcare services, lower socioeconomic status, and reduced dietary diversity among rural populations, which can increase nutritional vulnerability during TB illness. In addition, rural residents often face barriers to timely diagnosis and follow-up care, which may contribute to prolonged illness and further nutritional depletion.

Patients with extrapulmonary TB were more likely to be undernourished than those with pulmonary TB. Similar findings have been reported in a study conducted in India, where extrapulmonary disease was associated with lower BMI and a higher prevalence of undernutrition compared with pulmonary TB [23]. Extrapulmonary TB is frequently characterized by delayed diagnosis, prolonged disease duration, and multisystem involvement, which may contribute to increased metabolic demand and nutritional depletion [24].

In this study, undernutrition was more prevalent among patients with TB in the continuation phase of anti-TB treatment than in the intensive phase. This finding is similar to that of a study conducted in Addis Ababa [25]. This could be explained by the fact that patients who took anti-TB medications for up to 2 months anticipated beginning to recover from the illness when there were fewer episodes of nausea, vomiting, and appetite loss.

Poor treatment adherence was strongly associated with undernutrition in this study. This finding is consistent with evidence from other settings indicating that undernutrition and poor adherence frequently coexist and may adversely affect TB treatment outcomes [26]. Undernourished patients are more likely to experience drug-related side effects, reduced appetite, and physical weakness, which may interfere with consistent medication intake and engagement with treatment services [27]. The associations observed between treatment-related variables (such as treatment phase, duration, and adherence) and undernutrition are likely complex and potentially bidirectional. For instance, prolonged illness may lead to nutritional depletion, while undernutrition may delay recovery and affect treatment progression. These interrelationships should be interpreted cautiously.

Recent modeling studies have further highlighted the complex and dynamic relationships between socioeconomic conditions, nutritional status, and TB outcomes. For instance, mathematical and behavioral models have demonstrated how treatment-seeking behavior, income constraints, and nutritional status interact to influence disease transmission and treatment outcomes [[28], [29], [30], [31]]. These models emphasize that TB control is not solely a clinical issue but is also shaped by broader social and economic determinants. The findings of the present study are consistent with this perspective, underscoring the need for integrated interventions that address both medical and socioeconomic factors in TB care.

### *Strengths and limitations*

This study has several strengths. Standardized anthropometric measurements were used, enhancing the reliability of the nutritional status assessment. To our knowledge, this is the first study to assess the prevalence of undernutrition and its associated factors among adult patients with TB in this setting, thereby providing context-specific evidence to inform targeted nutritional interventions. In addition, multivariable analysis was employed to control for potential confounding factors. However, some limitations should be considered when interpreting the findings. First, due to the cross-sectional design, causal relationships cannot be established between undernutrition and the associated factors. The observed associations may be bidirectional; for example, poor treatment adherence may contribute to undernutrition, while undernutrition itself may reduce adherence due to physical weakness or drug-related side effects. Second, as an institution-based study, the findings may not be fully representative of all patients with TB in Somalia, particularly those who do not access healthcare services. This may introduce selection bias and limit the generalizability of the results. Finally, undernutrition was assessed solely using BMI, which does not capture micronutrient deficiencies or changes in body composition, such as muscle wasting. Consequently, the true burden of malnutrition may be underestimated, and caution is required when interpreting nutritional status based only on BMI.

## Conclusion

Undernutrition remains a significant challenge among adults with TB, affecting more than one-third of those receiving care. This study identified key sociodemographic and treatment-related factors associated with undernutrition. Based on the findings of this study, routine nutritional screening using BMI should be incorporated into TB care at the time of treatment initiation and during follow-up visits. Particular attention should be given to patient groups identified as being at higher risk of undernutrition. In addition, enhancing adherence support strategies and improving access to community-based and decentralized TB and nutrition services, particularly in rural settings, may help address nutritional vulnerability and support improved treatment outcomes.

## Declaration of competing interest

The authors have no competing interests to declare.
